# Investigating the effect of dehydromiltirone on septic AKI using a network pharmacology method, molecular docking, and experimental validation

**DOI:** 10.3389/fphar.2023.1145675

**Published:** 2023-03-15

**Authors:** Sijia Chen, Yanzhe Wang, Yuyuan Liu, Linnan Bai, Fengqin Li, Yue Wu, Xinmiao Xie, Nan Zhang, Chuchu Zeng, Ling Zhang, Xiaoxia Wang

**Affiliations:** ^1^ Department of Nephrology, Tongren Hospital, Shanghai Jiao Tong University School of Medicine, Shanghai, China; ^2^ Department of Nephrology, The Affiliated Suzhou Hospital of Nanjing Medical University, Suzhou Municipal Hospital, Suzhou, Jiangsu, China; ^3^ Department of Nephrology, Sir Run Run Shaw Hospital, Zhejiang University School of Medicine, Hangzhou, China; ^4^ Department of Obstetrics and Gynecology, Chengdu Women’s and Children’s Central Hospital, School of Medicine, University of Electronic Science and Technology of China, Chengdu, Sichuan, China

**Keywords:** septic AKI, network pharmacology, dehydromiltirone, COX2, apoptosis, mitochondrial dysfunction, inflammatory

## Abstract

Acute kidney injury (AKI) is a severe and frequent complication of sepsis that occurs in intensive care units with inflammation and rapid decline in renal function as the main pathological features. Systemic inflammation, microvascular dysfunction, and tubule injury are the main causes of sepsis-induced AKI (SI-AKI). The high prevalence and death rate from SI-AKI is a great challenge for clinical treatment worldwide. However, in addition to hemodialysis, there is no effective drug to improve renal tissue damage and alleviate the decline in kidney function. We conducted a network pharmacological analysis of *Salvia miltiorrhiza* (SM), a traditional Chinese medicine, which is widely used for the treatment of kidney disease. Then, we combined molecular docking and a dynamics simulation to screen for the active monomer dehydromiltirone (DHT) that has therapeutic effects on SI-AKI and investigated its potential mechanism of action through experimental validation. The components and targets of SM were obtained by searching the database, and 32 overlapping genes were screened by intersection analysis with AKI targets. GO and KEGG data showed that the functions of a common gene were closely related to oxidative stress, mitochondrial function, and apoptosis. The molecular docking results combined with molecular dynamics simulations provide evidence for a binding model between DHT and cyclooxygenase-2 (COX2), both of which are mainly driven by van der Waals interactions and a hydrophobic effect. *In vivo*, we found that mice pretreated with an intraperitoneal injection of DHT (20 mg/kg/d) for 3 days ameliorated CLP surgery-induced renal function loss and renal tissue damage and inhibited inflammatory mediators IL-6, IL-1β, TNF-α, and MCP-1 production. *In vitro*, the DHT pretreatment decreased LPS-induced expression of COX2, inhibited cell death and oxidative stress, alleviated mitochondrial dysfunction, and restrained apoptosis in HK-2 cells. Our research indicates that the renal preventive effect of DHT is related to maintaining mitochondrial dynamic balance, restoring mitochondrial oxidative phosphorylation, and inhibiting cell apoptosis. The findings in this study provide a theoretical basis and a novel method for the clinical therapy of SI-AKI.

## Introduction

Sepsis is a complex clinical syndrome that causes multiple organ dysfunction, and the kidneys are one of the most vulnerable organs and are prone to serious damage (Angus and van der Poll, 2013). Acute kidney injury (AKI) occurs in approximately 60% of sepsis patients, and the prognosis of patients with sepsis-induced AKI (SI-AKI) is poor, which is also a significant cause of high mortality (Bagshaw et al., 2007; Bellomo et al., 2017). Most SI-AKI survivors eventually develop chronic kidney disease (CKD), and complete loss of kidney function can occur in end-stage renal disease (Manrique-Caballero et al., 2021). Patients with a low quality of life and kidney failure enter the dialysis stage, which results in a large economic burden on society and has become a global public health problem requiring adequate attention (Hoste et al., 2018). The mechanisms of SI-AKI are complicated; inflammation, shock, microvascular dysfunction, and renal tubule injury have been confirmed in the pathophysiological processes of the disease (Gomez and Kellum, 2016). Reports suggest that an inflammatory storm and microcirculatory dysfunction can cause inadequate renal perfusion, and ischemia and hypoxia aggravate oxidative stress and NO release, which further causes renal tubular epithelial cell death and aggravates the deterioration of renal function (Fani et al., 2018). Nevertheless, there is a shortage of efficacious therapeutic strategies to improve the pathological damage of the kidneys and injury of tubular epithelial cells in SI-AKI other than hemodialysis (Rivers et al., 2001).

The kidneys are an extremely metabolic organ with the role of stabilizing circulating blood flow and maintaining electrolyte and acid-based balance (Carlstrom et al., 2015). The epithelial cells of renal tubules are the main sites of reabsorption and secretion and are rich in mitochondria (Che et al., 2014). As a center of energy metabolism and a major producer of ATP, the mitochondria ensure the survival of cell growth and the normal functioning of physiological processes (Martinez-Reyes and Chandel, 2020). Current studies confirm that mitochondrial dysfunction is an indispensable factor in the development of SI-AKI disease, and the central role of mitochondria-dependent apoptosis during cell death has been widely recognized in proximal tubule injury (Bock and Tait, 2020; Li et al., 2021). Inflammation and hypoxia lead to oxidative stress in cells, and the mitochondria are the main pro-apoptotic targets. Electron transport chain uncoupling blocks ATP production, and excess ROS accumulates in the cells (Nath and Norby, 2000). The apoptotic-related protein Bax is located in the cytoplasm and is transferred to the outer membrane of the mitochondria, which induces the opening of permeable pores in the mitochondrial double membrane, decreases membrane potential, and increases permeability (Jenner et al., 2022). The mitochondrial outer membrane breaks and releases cytochrome C to initiate the apoptotic process (Kalpage et al., 2020). In addition, extensive studies have shown that the inflammation-related factor cyclooxygenase-2 (COX2) regulates multiple physiological processes and is significantly increased in septic AKI, which suggests that COX2 may be a prospective target for SI-AKI (Zhao et al., 2016; Fan et al., 2021). COX2 is proven to be involved in the release of inflammatory mediators and control mitochondrial dynamics (Hua et al., 2015). Mitochondrial morphology is fragmented in the early stage of apoptosis, and the dynamic imbalance of the mitochondria is closely related to apoptosis. The overexpression of mitochondrial dynamin-related protein 1 (Drp1) raises the fission of the mitochondria and induces apoptosis and the knockdown of Drp1 inhibited apoptosis. Conversely, the overexpression of mitochondrial fusion protein 2 (Mfn2) protects cells from apoptosis (Nunnari and Suomalainen, 2012; Aranda-Rivera et al., 2021). In addition, energy metabolism disorders are another major manifestation of mitochondrial damage. The mitochondrial membrane potential decreases and the electron transport chain is blocked, which results in the inability to generate ATP, while ROS directly attacks cell DNA and other organelles causing cell apoptosis (Zhang et al., 2021). However, the mechanism of SI-AKI renal tubular epithelial cell apoptosis remains unclear, and further research is beneficial for discovering new molecular targets and providing innovative methods for clinical treatment.

*Salvia miltiorrhiza* Bge (SM) is a perennial erect herb of the Labiatae family, whose dried roots and rhizomes have been confirmed to have multiple pharmacological effects (XD et al., 2019). SM is a traditional Chinese medicine with multiple functions, including promoting blood circulation and having anti-inflammation and anti-oxidative stress effects (Su et al., 2015). The complex composition of SM mainly consists of water-soluble monomers represented by salvia phenolic acid and lipid-soluble monomers represented by tanshinone (XD et al., 2019). SM has been extensively applied in several clinical treatments of various diseases such as stroke (Kim et al., 2018), acute myocardial infarction (Li et al., 2018), and acute kidney injury (Chien et al., 2021). Studies have confirmed that various active monomers of SM can improve clinical symptoms by alleviating SI-AKI-induced kidney injury (Yuan et al., 2019). Dehydromiltirone (DHT) is a newly discovered monomeric component that protects the liver from acute injury by regulating p38 and the NF-κB signaling pathway in Kupffer cells (Yue et al., 2014). In this study, we found that DHT has a protective effect against SI-AKI, which not only results in more possibilities for drug development but also provides theoretical support for clinical treatment strategies.

From the perspective of the biological network, network pharmacology analyzes the complex system of traditional Chinese medicine and contains an interaction between the complex ingredients of Chinese herbs and a biological network and the interaction pattern (Zhou et al., 2020). It is a new model that can be used to estimate the relevance between multiple drug targets and the disease network to analyze the network properties *via* the association and connection of knots in a biological network (Zhang et al., 2019). In this study, we constructed a “disease–gene target–drug” network by screening for the targets of SM for AKI treatment and searched for active ingredients and molecular targets. This is combined with an experimental validation to investigate the beneficial effect and potential mechanism of DHT on SI-AKI *in vitro* and *in vivo*. This network pharmacology study is helpful in finding, optimizing, and confirming molecular targets and further elucidates the action mechanism of medicine and provides important guidance for the discovery of new drugs.

The purpose of the research is to explore the function and possible target of DHT on SI-AKI. Through network pharmacology, molecular docking, and molecular dynamics simulation combined with experimental verification, we found that the protection of DHT against SI-AKI may be associated with the downregulation of COX2 to inhibit mitochondrial dysfunction and improve apoptosis.

## Materials and methods

### Bioactive compounds of SM and the collection of related targets

SM component information was obtained by searching the TCMSP (http://tcmspw.com/) and TCMID databases (http://www.megabionet.org/tcmid/), and possible drug small molecules were screened using ADME parameters (Yamashita and Hashida, 2004) including drug-likeness (DL), oral bioavailability (OB), and half-life (HL). In this study, the threshold values of OB ≥ 20% and DL ≥ 0.1 were utilized for herbal component screening. Based on the BATMAN (http://bionet.ncpsb.org/batman-tcm/) and TCMSP databases, the corresponding target proteins of chemical small molecules were predicted, and a preliminary pharmacological network of herb–molecule–target proteins was constructed.

### AKI-related gene acquisition

A search was conducted in the CTD (http://ctdbase.org/) and DisGeNET (version6.0, http://www.disgenet.org/web/DisGeNET/menu/home/) databases using the key term “Acute kidney injury” to obtain genes concerned with AKI, and those with an inference score of 60 or more were intersected with the predicted target proteins in the previous step. This was conducted for screening to narrow down the target proteins, and the intersection between drug targets and genes related to acute kidney injury was integrated for follow-up verification.

### Gene Ontology and the Kyoto Encyclopedia of Genes and Genome Pathway Analysis

Based on previous methods (Liu et al., 2021), the clusterProfiler package was utilized to assess GO functional enrichment of intersecting targets, including molecular functions (MF), biological processes (BP), and cellular components (CC), and the same approach was applied to KEGG enrichment analysis. Significant results were determined using a *p*-value ≤ 0.05, and bubble plots were visualized to show the results of enrichment.

### Protein–protein interaction (PPI) analysis

A STRING database (version10.0, http://www.string-db.org/) was performed to estimate the PPI of the target proteins. Required confidence (combined score) > 0.4 was chosen as the threshold of protein–protein interaction. A network diagram of the PPI relationship was generated using Cytoscape. The top 10 key targets were selected by calculating the MCC (maximal clique centrality) score through the cytoHubba plugin.

### Herb–ingredient–target–pathway network construction

The results were imported of the active ingredients, intersecting targets, and enrichment pathways into Cytoscape software for visual analytics, and pharmacological networks were constructed to show the pathway regulation mechanism of relevant targets of ingredients in the remedy. The color and shape of the nodes were adjusted to derive the “herb–ingredient–target–pathway” network diagram.

### Molecular docking and molecular dynamics simulation

The PDB database was used to obtain the crystal structure of COX2, and the PubChem database was used to gain the 3D structure of DHT. The energy is minimized in the MMFF94 force state, which is implemented using Chem3D 14.0 software. Charge was added to all receptor proteins, the atomic types were distributed and dehydrated, and the original ligands and ions were extracted. All processes were realized by PyMOL 2.5.2. The docking box center, according to the location of the active site, was defined by the PyMol plugin center_of_mass.py. The standard box length on the box side was adjusted to 22.5 Å. To obtain the form of PDBQT required for AutoDock Vina 1.1.2 docking, all treated receptor proteins and small molecules were converted using ADFRsuite 1.02. Visualization results were processed using PyMol software. To investigate the dynamic interactions of DHT and COX2, molecular dynamics simulations were carried out using AMBER 18 software. The Hartree–Fock (HF) SCF/6–31G* and antechamber module of Gaussian 09 software were performed to calculate the charge of DHT. Target and active components were expressed by an ff14SB protein force field and GAFF2 small-molecule force field, respectively, and hydrogen atoms were placed into each system. At 10 Å from the system, the sectional octahedral TIP3P solvent tank was penetrated. The LEaP module was used to insert hydrogen atoms into each system, and a truncated octahedral TIP3P solvent box was added 10 Å away from the system. In addition, Na^+^/Cl^−^ was inserted to balance the charge of the system, and the parameter file and topology for the simulation were finally exported. The system energy was optimized before simulation, and a 2500 step conjugate gradient method and a 2500 step steepest descent method were performed (Mark and Nilsson, 2002). After energy optimization, the system temperature was slowly increased from 0 K to 298.15 K at a constant heating rate and fixed volume. The solvent molecules were evenly spread in the solvent tank, and the isothermal isomer system was simulated at a system maintenance temperature of 298.15 K for 500 ps. Finally, the entire system was equilibrium simulated for 500 ps. Two complexes were simulated with a 50 ns isothermal isobaric system under a periodic boundary condition.

### Binding free energy calculation

To accurately evaluate the binding mode of DHT and COX2, our study further calculated the binding energy of DHT and COX2 using the MM/GSBA method (Genheden and Ryde, 2015) based on the molecule dynamics trajectory of 45–50 ns. The free energy was calculated according to the formula (Kollman et al., 2000):

$${\Delta G}_{bind}={\Delta G}_{complex}–\;\left({\Delta G}_{receptor}+{\Delta G}_{ligand}\right),$$

$$={\Delta E}_{internal}+{\Delta E}_{VDW}+{\Delta E}_{elec}{+\Delta G}_{GB}+{\Delta G}_{SA.}$$

In this formula, ΔEinternal denotes internal energy, ΔE_VDW_ denotes van der Waals interaction, and ΔE_elec_ denotes an electrostatic interaction. Bond energy (ΔE_bond_), angular energy (ΔE_angle_), and torsional energy (ΔE_torsion_) are collectively called internal energy. ΔG_GB_ and ΔG_SA_ are uniformly called solvation free energy. The polar solvation free energy is represented by ΔG_GB_, and the non-polar solvation free energy is represented by ΔG_SA_.

### SI-AKI model and treatment

For this study, 8–10-week-old C57BL/6 mice (male, pathogen-free) were purchased from the Model Animal Research Center of Nanjing University (Nanjing, China). Consistent with previous reports (Rittirsch et al., 2009), a septic AKI animal model was built by cecal ligation and puncture (CLP) surgery. Mice were anesthetized with 2% isoflurane induction and 1.4% isoflurane maintenance, the cecum was silk-ligated and pierced twice by a 20-gauge needle, and after closing the abdomen, saline was injected subcutaneously for fluid resuscitation. Blood and kidney samples were harvested after 24 h. A 20 mg/kg/d dose of DHT was injected intraperitoneally into the mice for three consecutive days prior to CLP surgery. The sham group received identical dissection compared to the mice in the CLP group but without cecum ligation and puncture.

### Renal function

After euthanasia of all animals, blood samples were collected from the eyes of the mice and serum was separated to evaluate the renal function. BUN and Scr were assessed using an automated biochemical analyzer (Chemray 800, Rayto, Shenzhen, China).

### Hematoxylin-eosin staining

The mouse kidney tissue was placed into 4% paraformaldehyde for fixation, then the tissue was dehydrated with gradient ethanol, paraffin was used to embed the tissue, and the kidneys were cut into 2–3 μm sections. Renal tubular morphology was detected under a microscope after staining with hematoxylin and eosin. The percentage of damaged tubules was calculated based on the dilatation of the tubules, the loss of the brush border, and the formation of tubular casts to evaluate the tubular damage.

### Cell culture and treatment

Human renal proximal tubular cells (HK-2 cells) were obtained from the Chinese Academy of Sciences for the *in vitro* experiments. HK-2 cells were cultured at 37°C, 5% CO_2_, and 95% O_2_ in a humidified atmosphere and fostered in DMEM/F12 with 10% FBS and 1% penicillin/streptomycin. A measure of 5 μg/ml lipopolysaccharide (LPS) (Sigma-Aldrich, USA) was added to evaluate the HK-2 cell damage of SI-AKI *in vitro*. Cells were pretreated with 5 μM DHT for 2 h and then co-cultured with LPS for 24 h. NS-398 (5 μM, Cayman Chemical, USA) was used to selectively inhibit COX2 in the HK-2 cells.

### Quantitative RT-PCR

Total RNA was extracted from the kidney with TRIzol reagent (Takara, Tokyo, Japan). The primer was synthesized from Sangon Bioengineering Technology Co., Ltd. (Shanghai, China). The mRNA concentration was measured using NanoDrop 8000 (Thermo Fisher Scientific, USA) and synthesized cDNA *via* a PrimeScriptTM RT Reagent Kit (Takara, Kyoto, Japan). Quantitative RT-PCR was implemented using the QuantStudio 3 qRT-PCR system (Thermo Fisher Scientific, CA). The primer sequence is indicated in Supplementary Table S1. The relative target mRNA levels were standardized with *β*-actin.

### Cell viability

The viability of cells was tested using a cell counting kit-8 (CCK-8, Beyotime, China) assay. Briefly, cells were fostered at a density of 1 × 10^4^ cells/well in a 96-well plate. After overnight cell adherent growth, they were disposed and grouped as described previously, and 10 μL of CCK-8 reagent was added per well. The 96-well plate was then placed in an incubator at 37°C for 20 min protected from light. Each well optical density (OD) value at 450 nm was tested through a microplate reader (Thermo Fisher Scientific, USA). The CCK-8 assay was used to detect the IC_50_ value of DHT.

### ROS detection

The content of ROS in the cell cultures was determined using oxidation-sensitive dye 20,70-dichlorofluorescein diacetate (DCFH-DA). HK-2 cells were seeded in six-well plates (1×10^4^ cells/well) and then arranged and grouped as described previously. After washing the cells twice in PBS, the DCFH-DA (Beyotime, China) was diluted 1,000 times in a serum-free DMEM/F12 medium. Then, at least 1 ml of dye solution was placed in six-well plates, and the staining process was protected from light for 20 min at 37°C. After washing three times with serum-free medium, the intensity of the fluorescence in each group was measured using a fluorescence microscope (Nikon, Japan).

### Western blot

After washing twice with precooled PBS, proteins were extracted from HK-2 cells by adding protein lysis buffer (Beyotime, China). The cells in each group were scraped and homogenates were transferred to a centrifugal tube, all tubes were placed on ice for 30 min to allow the cells to fully lyse, and this was followed by a 15-min centrifugation at 12000r/min and 4°C. The total proteins were separated sequentially by SDS polyacrylamide gel electrophoresis for the different sizes of proteins. After electrophoresis, the proteins were transmigrated from the gel onto a PVDF membrane. The membrane was blocked with 5% skim milk for 1h, and the protein bands were incubated overnight with the corresponding primary antibody solution in a refrigerator at 4°C. The primary antibody information is as follows: COX2 (ab283574, Abcam, 1:1,000), Drp1 (12957-1-AP; Proteintech, 1:2000), Mfn2 (12186-1-AP; Proteintech, 1:2000), Bcl2 antibody (ab59348; Abcam, 1:1,000), Bax (ab32503; Abcam, 1:1,000), and *β*-actin (AF5006; Beyotime, 1:1,000). The secondary antibody sheep anti-rabbit immunoglobulin G (IgG) (A0208, Beyotime, 1:1,000) was incubated with protein bends for 1 h at room temperature. Thereafter, TBST is used to wash the protein bends to keep the background clean, and the membrane is then immersed in ECL reagent to detect the signals of the blot. The quantification of each protein band gray value was performed using ImageJ software. *β*-Actin serves as an internal control, and the relative expression of target protein was calibrated by the gray value of the target band divided by the gray value of the *β*-actin band. Each group was normalized with the control group to obtain the fold change.

### Seahorse analysis

The HK-2 cell mitochondrial respiration function was evaluated using the XFe24 Seahorse Analyzer (Agilent Technologies). The real-time oxygen consumption by the mitochondria in HK-2 cells was assessed under specific activators and inhibitors of the mitochondria. HK-2 cells were seeded into 24-well cell culture microplates (1.5×10^4^ cells/well). To maintain natural sedimentation, the microplates were transferred to the incubator after being placed on an ultraclean table for 1 h. HK-2 cells were grouped and treated as described previously. The probe plate was hydrated overnight in a CO_2_-free 37°C incubator 1 day before mitochondrial respiration oxygen consumption rate (OCR) detection. The next day, the growth medium was removed in the cultured plate before the experiment, and the cells were gently washed three times with the XF Base Medium at pH 7.4 prepared from 2 mM glutamine, 1 mM pyruvate, and 10 mM glucose. Then, the cultured cells with the buffer were placed in a CO_2_-free incubator at 37°C for 60 min. At the same time, the solution was configured according to the concentration of the reagent in the pre-optimized Mitochondrial Pressure Test Kit (Agilent) that had the greatest effect on mitochondrial respiration as follows: using 1.5 µM oligomycin (complex V inhibitor) to detect the ATP turnover rate and proton leakage, 1 µM FCCP (a respiratory uncoupler) was used to determine maximal respiratory function. The reserve capacity is equal to the maximal OCR minus basal respiration, and 0.5 µM rotenone/antimycin A (inhibitor of complex I and complex III) was injected to completely shut down mitochondrial respiration. Then, the microtiter plates were examined in an XFe24 analyzer. OCR was recorded over time, and the parameters of mitochondrial function were calculated, including mitochondrial basal respiration, proton leakage, spare capacity, and maximal respiration.

### Mitochondrial membrane potential (MMP) detection

The MMP in different groups was measured using a JC-1 staining kit (Beyotime, China). HK-2 cells (1×10^4^ cells/well) were already prepared in six-well plates and cultured in an incubator. After treatment, as mentioned previously, PBS was used to wash the cells with poor adhesion status, and JC-1 staining was conducted based on the instructions. Then, 1 ml of precooled JC-1 buffer solution was replaced for each well to wash away the unbound probes and this was repeated twice. The changes in MMP were recorded using a fluorescence microscope (Nikon, Japan).

### Statistical analysis

GraphPad Prism 7 (GraphPad Software, Inc.) was used to perform the statistical analysis and to create graphs. Data are presented as the mean ± SE. A comparison between the two groups was conducted using the Student’s t-test. The one-way ANOVA was chosen for statistical comparison of multiple groups. A *p*-value < 0.05 was considered statistically significant.

## Results

### Potential targets of SM in the treatment of AKI and functional enrichment analysis

**FIGURE 1:**
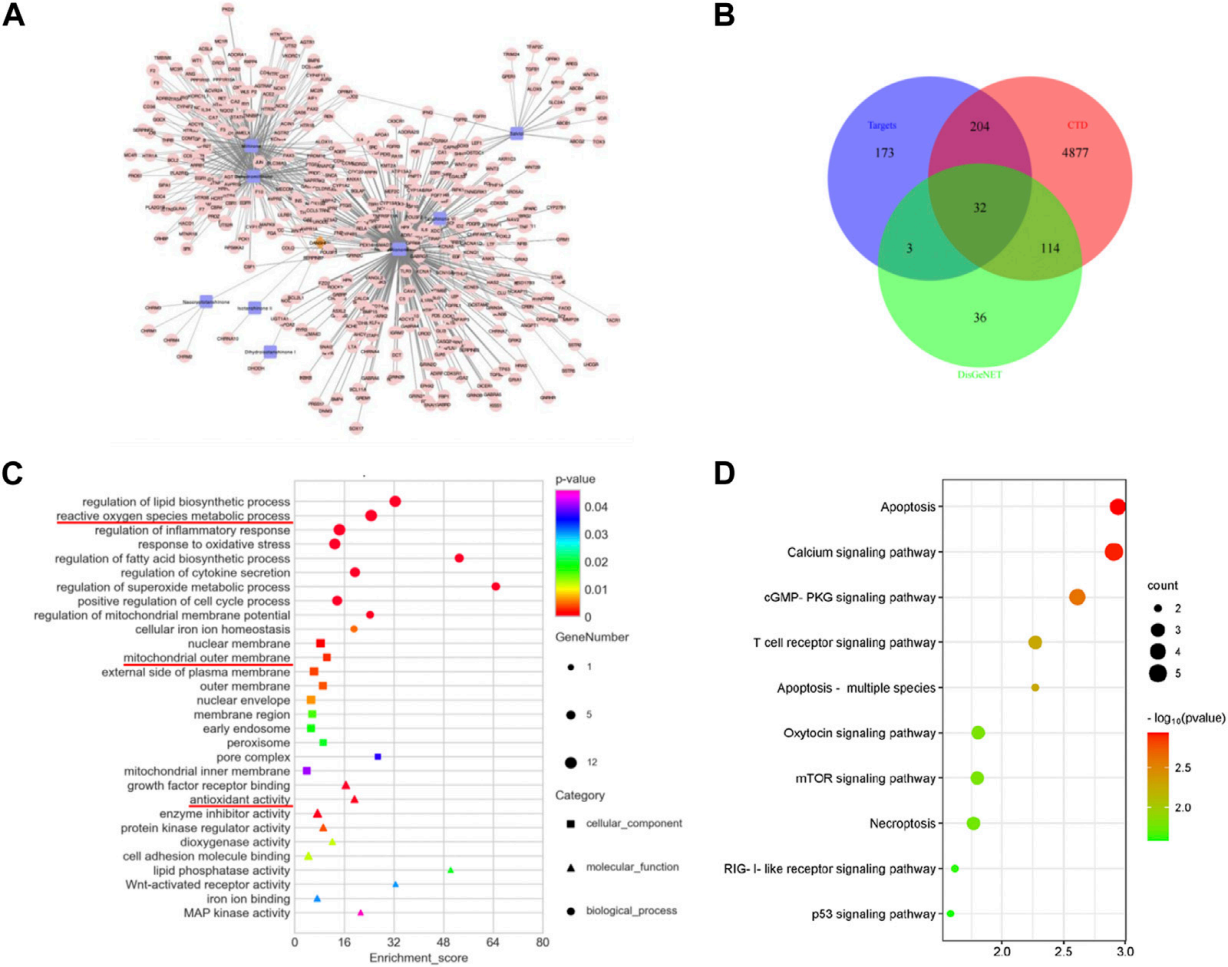
Overlapped targets and functional enrichment analysis between SM and AKI. **(A)** Target prediction and network analysis of SM components. A total of nine key molecules were screened. The brown diamond represents SM, the blue square represents the components, and the pink dot represents the targets. **(B)** Venn diagram of the intersection genes among SM targets and AKI targets from the DisGeNET and CTD database. **(C)** GO enrichment analysis. The square represents the cellular component, the triangle represents the molecular function, and the circle represents biological process. **(D)** KEGG enrichment analysis. The vertical axis represents the pathway name, and the horizontal axis represents the number of enriched genes.

A search of the TCMSP and TCMID databases was conducted using the keyword “*Salvia miltiorrhiza*” to obtain information of the active ingredients, and the ADME parameters were used to screen the possible drug small molecules with the inclusion criteria of OB ≥ 20% and DL ≥ 0.1. Using the TCMSP and BATMAN databases to predict the corresponding target proteins of chemical small molecules based on the ranking of potential target interactions and according to their similarity to known targets, a total of nine active compounds and 412 compound-related target proteins were obtained (Figure 1A). Meanwhile, 412 target proteins were intersected with the genes with an inference score >60 in the CTD and the acute kidney injury-related genes in the DisGeNET database, and finally, 32 intersection-related targets were obtained and the results are presented in the form of the Venn diagram (Figure 1B). To further explore the biological functions of the overlapped genes, GO and KEGG enrichment analyses were conducted. The GO analysis data indicate that the common target is related to the metabolic process of reactive oxygen species, inflammatory response regulation, oxidative stress response, mitochondrial membrane potential regulation, the mitochondrial outer membrane, and antioxidant activity (Figure 1C). An analysis of KEGG pathway enrichment was performed using the clusterProfiler for intersection target protein genes. The results show that intersection targets were mainly focused on apoptosis pathways related to cell survival (Figure 1D).

### Protein–protein interaction (PPI) analysis and “herb–ingredient–target–signal pathway” network construction of SM

**FIGURE 2:**
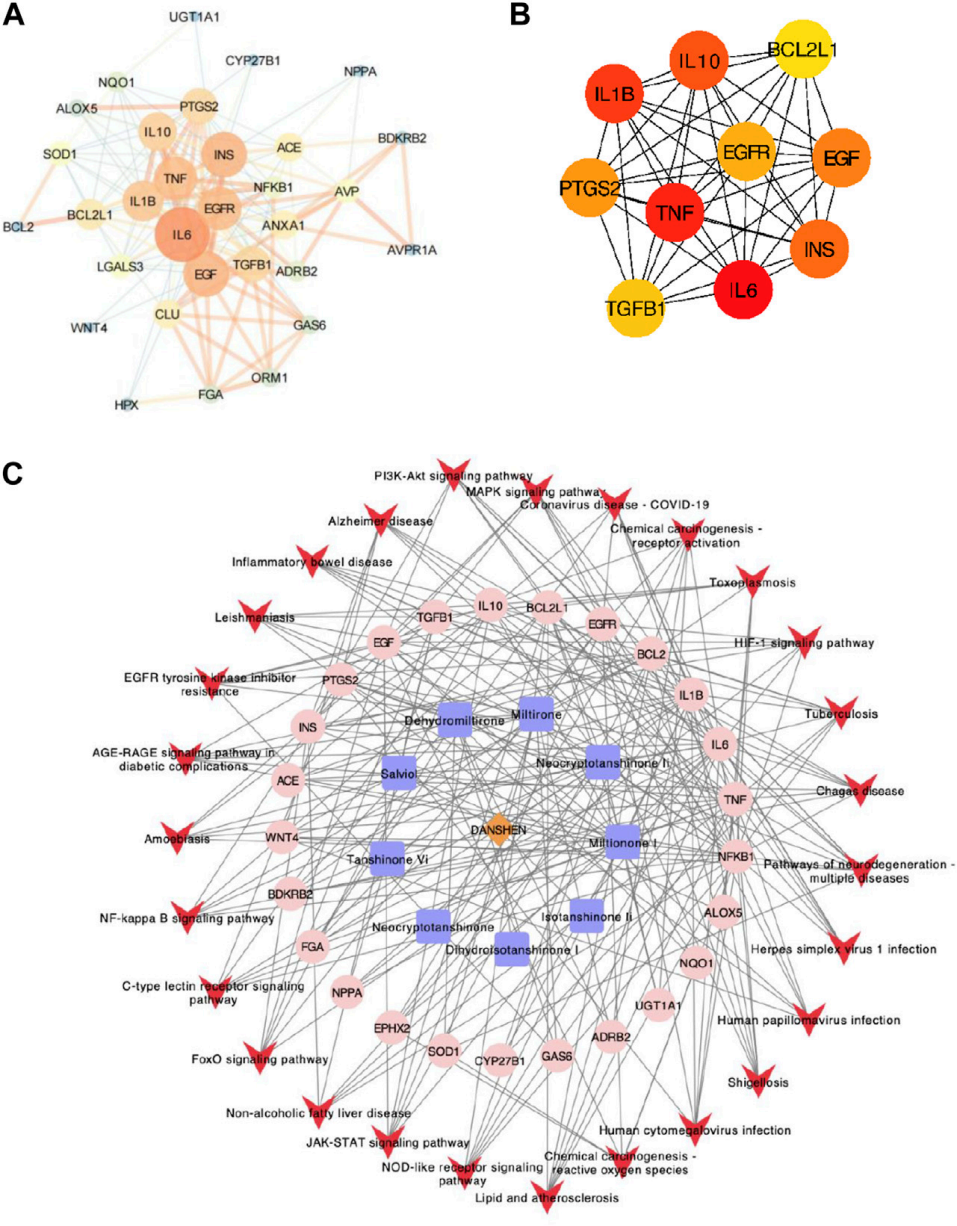
PPI network analysis. **(A)** PPI network analysis of 32 intersecting genes; the dot size represents the degree value, and the line thickness represents the correlation degree. **(B)** PPI network analysis of the top 10 potential hub genes. **(C)** Herb–ingredient–target–signal pathway network. The brown diamond represents the herb, the blue square represents components, the pink dot represents related targets, and the red arrow represents the pathway.

Further exploration of the interaction relationship of overlapped genes was conducted through PPI analysis. A threshold value of requested confidence (combined score) > 0.4 was required for the protein–protein interaction relationship, and the results showed that 31 proteins had interactions (Figure 2A). A network graph was constructed for all interactions using Cytoscape, and an MCODE clustering analysis was executed, which included 157 relationship pairs. To find the potential hub genes, 32 intersecting proteins were screened for the top 10 key targets by calculating the MCC (maximal clique centrality) scores including IL-6, TNF, IL-1β, IL-10, INS, EGF, PTGS2 (also known as COX2), EGFR, TGFβ1, and BCL2l1 (Figure 2B). Based on the herb–component–target protein–pathway, the pharmacological network was finally constructed by selecting the herbs and molecules related to the intersection target and the pathways with more than five enriched gene entries, which consisted of 59 nodes and 242 relationship pairs, including one herb node, nine chemical component nodes, 25 target protein nodes, and 27 pathway nodes (Figure 2C).

### Molecular docking models between active ingredients and potential targets

**FIGURE 3:**
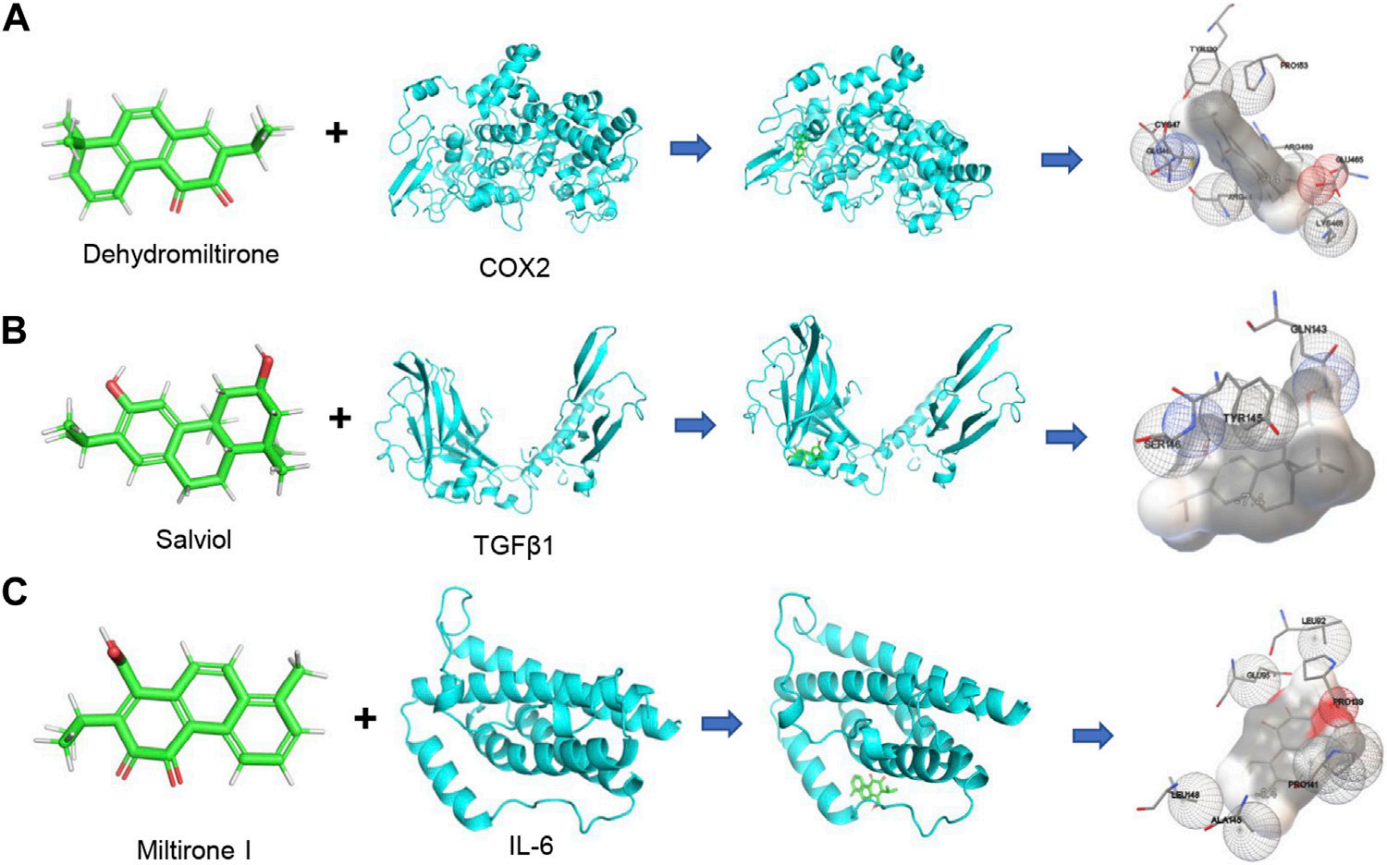
Diagram of molecular docking models between potential targets and core components. **(A)** Dehydromiltirone–COX2. **(B)** Salviol–TGF-β1. **(C)** Miltirone I–IL-6. The hydrogen atoms in the receptor are in close contact with atoms in the ligand that are shown as spheres.

The combination of small molecules and targets was simulated by molecular docking to evaluate the interactions. The PubChem Compound provides the 3D molecular structure of the ingredients, and the molecular structure of the targets was retrieved from the PBD database (https://www.rcsb.org/). AutoDock Vina software was used to analyze the local binding sites for analysis, and molecular docking simulations were performed. The affinity was calculated using a Lamarckian genetic algorithm (LGA), a docking fraction less than -5 indicated good binding, and the smaller the fraction the better the docking result. Based on the previous results, we analyzed the interaction between central targets and compounds. The top three docking models of affinity included dehydromiltirone–COX2 (Figure 3A), salviol–TGF-β1 (Figure 3B), and miltirone I–IL-6 (Figure 3C). The molecular docking results suggested that DHT and COX2 have the highest affinity score (Supplementary Table S2).

### Verification of the DHT and COX2 binding pattern

**FIGURE 4:**
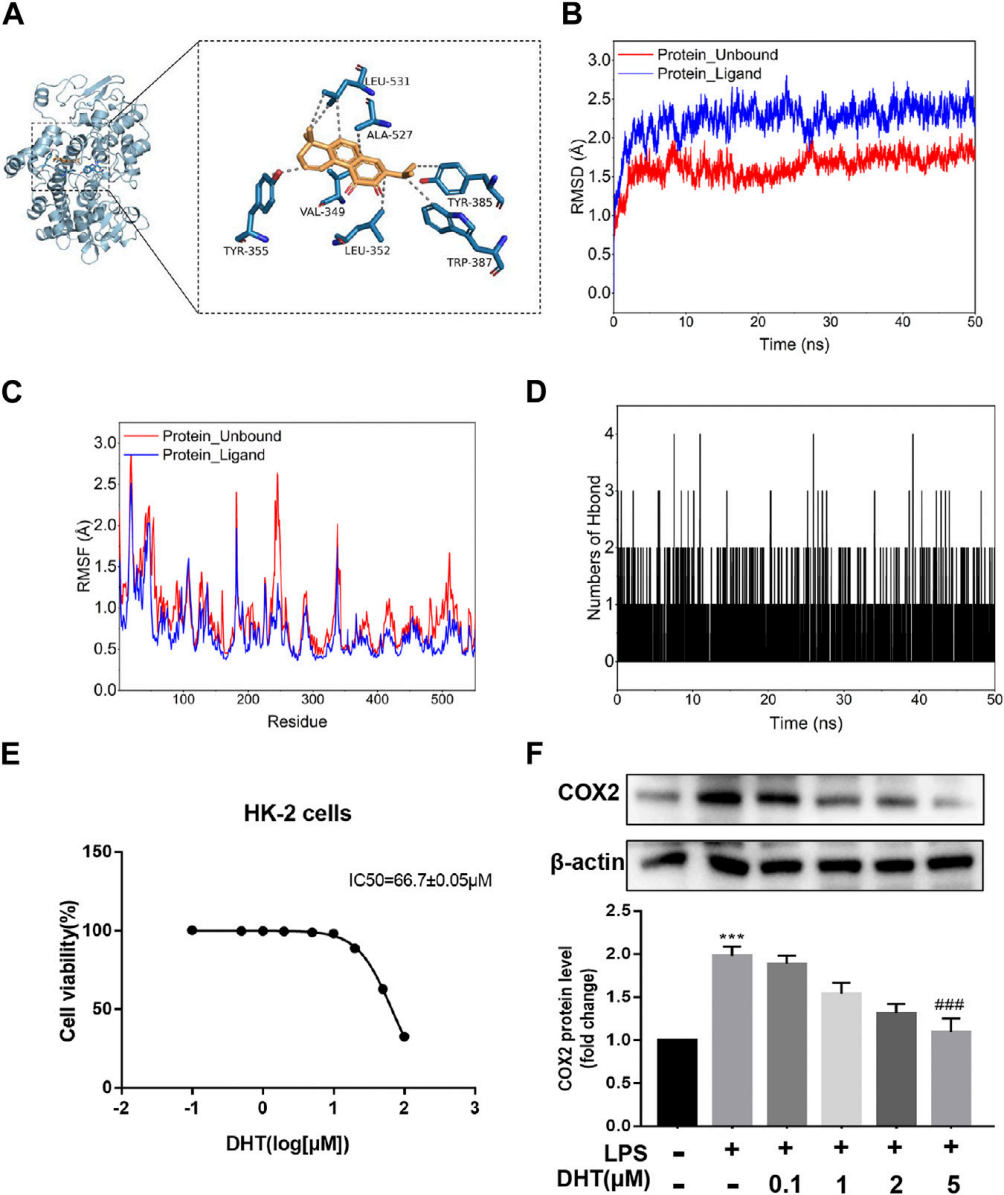
Docking pattern and molecular dynamics simulation of DHT binding to COX2. **(A)** Binding model analysis of DHT and COX2. The left panel shows the overall view, while the right panel shows the local view. The orange stick represents DHT, the blue picture represents COX2, and the gray dotted line represents the hydrophobic effect. **(B)** RMSD of the DHT and COX2 complex with time in the molecular dynamics simulation. **(C)** RMSF of the DHT and COX2 complex based on the molecular dynamics simulation. **(D)** Changes in the number of hydrogen bonds between small molecules and proteins during the molecular dynamics simulations. **(E)** Cell viability was measured by performing a CCK-8 assay after incubating HK-2 cells at different concentrations of DHT for 24 h. The IC_50_ value of DHT is 66.7 ± 0.05 μM. **(F)** Representative Western blot and quantitative data showing different concentrations of DHT on COX2 expression in LPS-induced HK-2 cells (n = 3). ^***^ *p* < 0.001 vs. Con, ^###^ *p* < 0.001 vs. LPS.

A follow-up validation was conducted for dehydromiltirone–COX2. The docking mode showed that DHT was bound in the internal active pocket of the COX2 protein, which was the active site as determined by the crystal structure. The results showed that DHT interacted with Leu-352, Leu-531, Tyr-355, Ala-527, Tyr-385, Trp-387, and Val-349 on the COX2 protein (Figure 4A). Next, a molecular dynamics (MDS) simulation was performed to explore the binding stability and determine the accurate binding energy between DHT and COX2. The movement process of the complex was characterized by the root mean square deviation (RMSD). The two systems were set up with or without DHT converge in the first 5 ns of the simulation, which indicates that the system can quickly move toward a stable movement state. The RMSD was higher for the system with bound small molecules at stable fluctuations, which indicated better binding of DHT and COX2 (Figure 4B). The flexibility of the proteins during molecular dynamic simulations was represented by the root mean square fluctuation (RMSF). Compared with the unbound system, the RMSF in the bound system was smaller, which suggests that DHT could partially stabilize the structure of the COX2 protein (Figure 4C).

The calculation results of the MM-GBSA method show that the binding energy of DHT and COX2 was -36.73 ± 2.37 kcal/mol (Figure 4D). These data indicate that the binding affinity of DHT/COX2 is very strong. The van der Waals energy is the main contribution, while electrostatic energy plays a secondary role. A quantity variance was detected in the hydrogen bond formation during the process, and the results suggest that the number of formed hydrogen bonds fluctuated between 0 and 4 and remained at 0–1 hydrogen bonds most of the time (Table 1). This indicates that hydrogen bonds are formed less during the binding of the complex system, and hydrophobic interactions may play a major driving force in the connection of DHT and COX2. HK-2 cells were treated with different concentrations of DHT for 24 h, and the CCK-8 assay was used to detect the cell survival rate. The results show that the IC_50_ value of DHT was 66.7 ± 0.05 μM (Figure 4E). Then, we verified COX2 expression in the LPS-induced HK-2 cells pretreated with or without DHT. Compared with the control group, Western blot results indicated that COX2 was upregulated in HK-2 cells treated with LPS, whereas different concentrations of DHT (0.1 μM, 1 μM, 2 μM, and 5 μM) pretreatment reversed this performance. Overall, DHT depressed LPS-induced COX2 expression in a dose-dependent manner (Figure 4F). These data suggest that DHT may exert pharmacological effects by binding to COX2 to promote degradation.

**TABLE 1:** Energy components and combined free energy are calculated using the MM/GBSA method.

System name	Dehydromiltirone/COX2
ΔG_bind_	-36.73 ± 2.37
ΔE_VDW_	-43.40 ± 2.02
ΔE_elec_	3.45 ± 1.79
ΔG_GB_	8.69 ± 1.27
ΔG_SA_	-5.47 ± 0.11

ΔG_bind_, binding free energy; ΔE_VDW_, van der Waals energy; ΔE_elec_, electrostatic energy; ΔG_GB_, electrostatic contribution to solvation; ΔG_SA_, non-polar contribution to solvation.

### DHT pretreatment alleviates CLP-induced acute kidney injury in mice

**FIGURE 5:**
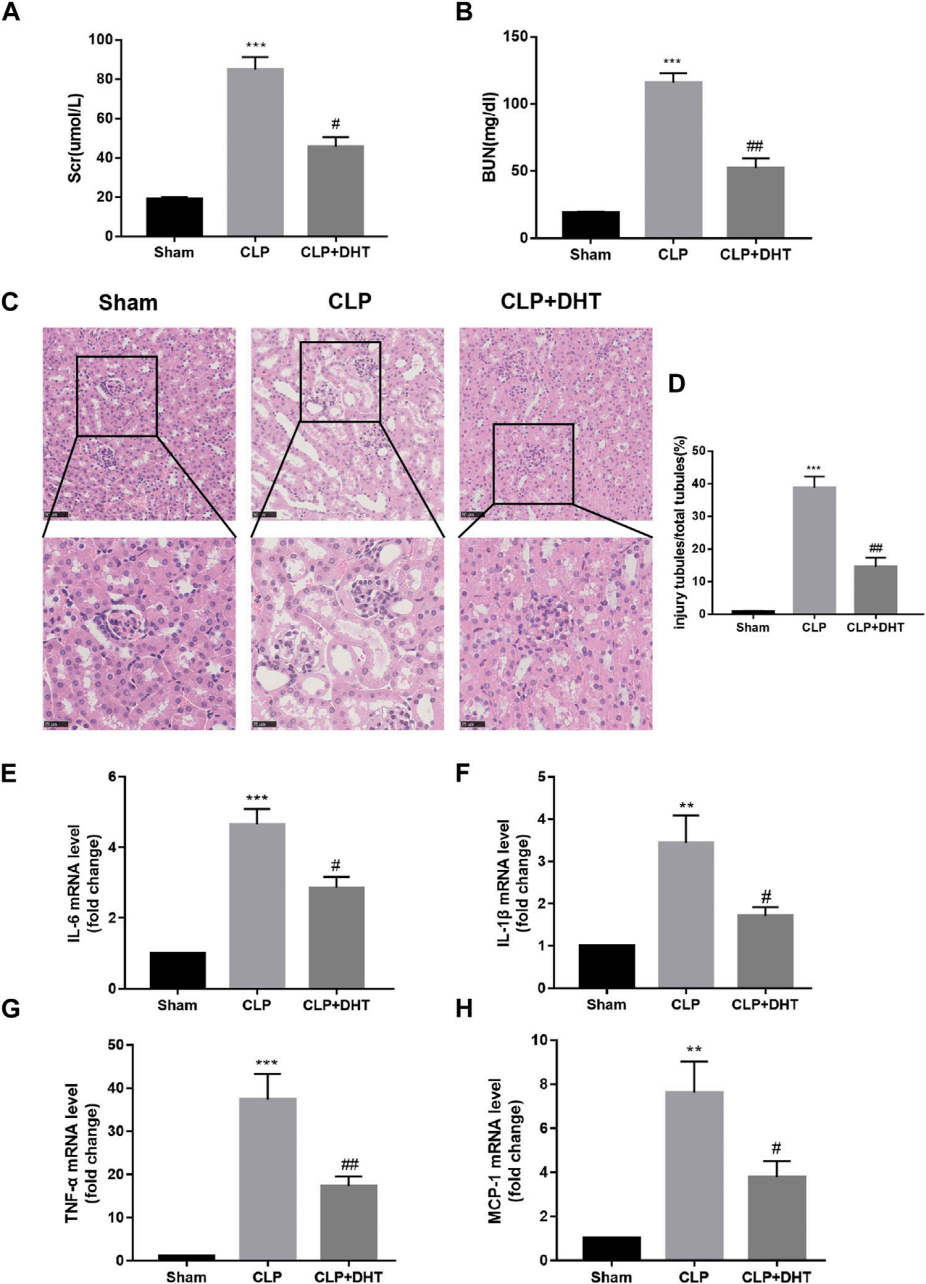
DHT protects against CLP-induced AKI. **(A,B)** Comparison of Scr and BUN levels in sham, and CLP-induced AKI with or without DHT treatment (n = 3). ^***^ *p* < 0.001 vs. sham, ^#^ *p* < 0.05, ^##^ *p* < 0.01 vs. CLP. **(C)** HE staining of kidney tissue in each group. Renal tubular dilatation, loss of tubular brush border, and cast formation were observed in CLP-induced AKI, while DHT pretreatment partially alleviated those changes. Scale bars: 50 μm (upper panel), 25 μm (lower panel). **(D)** Quantitative analysis of injured tubules in each group (n = 6). ^***^ *p* < 0.001 vs. sham, ^##^ *p* < 0.01 vs. CLP. **(E–H)** mRNA expression levels of IL-6, IL-1β, TNF-α, and MCP-1 were determined by qRT-PCR (n = 3). ^**^ *p* < 0.01, ^***^ *p* < 0.001 vs. sham, ^#^ *p* < 0.05, ^##^ *p* < 0.01 vs. CLP.

To clarify the influence of DHT on SI-AKI, we pretreated mice with an intraperitoneal injection of DHT (20 mg/kg/d) 3 days in advance, and then the mice were processed through the CLP operation to establish the septic-AKI mouse model. Kidney function-related Scr and BUN index levels were tested to estimate the degree of injury, and the results showed that Scr and BUN levels were elevated in mice under CLP surgery compared with the sham group, while DHT pretreatment partially alleviated the decrease in renal function (Figures 5A, B). The renal histological changes were observed in detail by HE staining. The data suggest that CLP surgery resulted in a loss of brush border, renal tubular dilatation, and tubular cast formation. In contrast, renal tubular injury was partially attenuated in DHT pretreated CLP mice (Figures 5C, D). Inflammation also plays a significant role in kidney injury subjected to sepsis. qRT-PCR was conducted to measure the production of pro-inflammatory mediators. These data suggest that CLP surgery induced the IL-6, IL-1β, TNF-α, and MCP-1 mRNA levels in the mice kidneys, while DHT pretreatment reversed these changes (Figures 5E–H). This suggests that the pretreatment with DHT partially alleviates septic AKI in mice.

### DHT suppressed cell death and oxidative stress in HK-2 cells subjected to LPS

**FIGURE 6:**
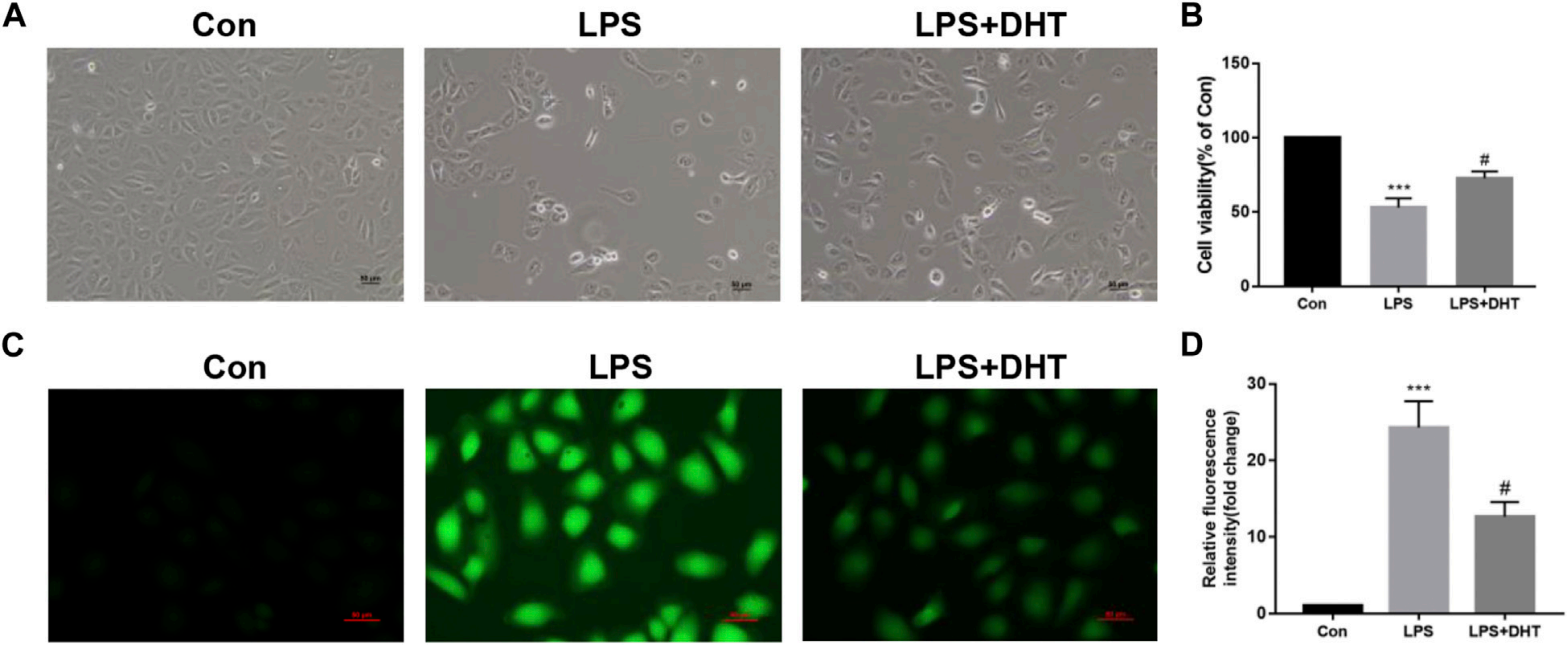
Effect of DHT on cell viability and oxidative stress in LPS-induced HK-2 cells. **(A)** Representative phase contrast images of HK-2 cell morphology in each group. Scale bars: 50 μm. **(B)** Cell viability was determined using a CCK-8 assay (n = 3). ^***^ *p* < 0.001 vs. Con, ^#^ *p* < 0.05 vs. LPS. **(C)** Representative image of ROS measured by fluorescence microscopy in each group. Scale bars: 50 μm. **(D)** Quantitative data of ROS fluorescence intensity in HK-2 cells (n = 3). ^***^ *p* < 0.001 vs. Con, ^#^ *p* < 0.05 vs. LPS.

To evaluate the role of DHT on LPS-induced HK-2 cells, we pretreated cells with 5 μM DHT for 1 h and then co-cultured them with LPS (5 μg/ml) for 24 h. The morphological results of cells under an inverted microscope showed that DHT pretreatment reversed the growth restriction and morphological changes of HK-2 cells after LPS (Figure 6A). CCK-8 was conducted to assess the cell activity. Compared with the control group, LPS caused a significant reduction in the viability of cells, but cell death was partially alleviated by DHT preconditioning (Figure 6B). To examine whether DHT protects renal tubular epithelial cells from oxidative stress induced by LPS, we evaluated the production of intracellular ROS, which is a key indicator of oxidative stress. Intracellular ROS production increased with exposure to LPS, while DHT improves oxidative stress by inhibiting ROS production (Figures 6C, D). These data demonstrate that DHT can reverse ROS accumulation and cell death induced by LPS *in vitro*.

### DHT protects HK-2 cells from LPS-induced mitochondrial dysfunction

**FIGURE 7:**
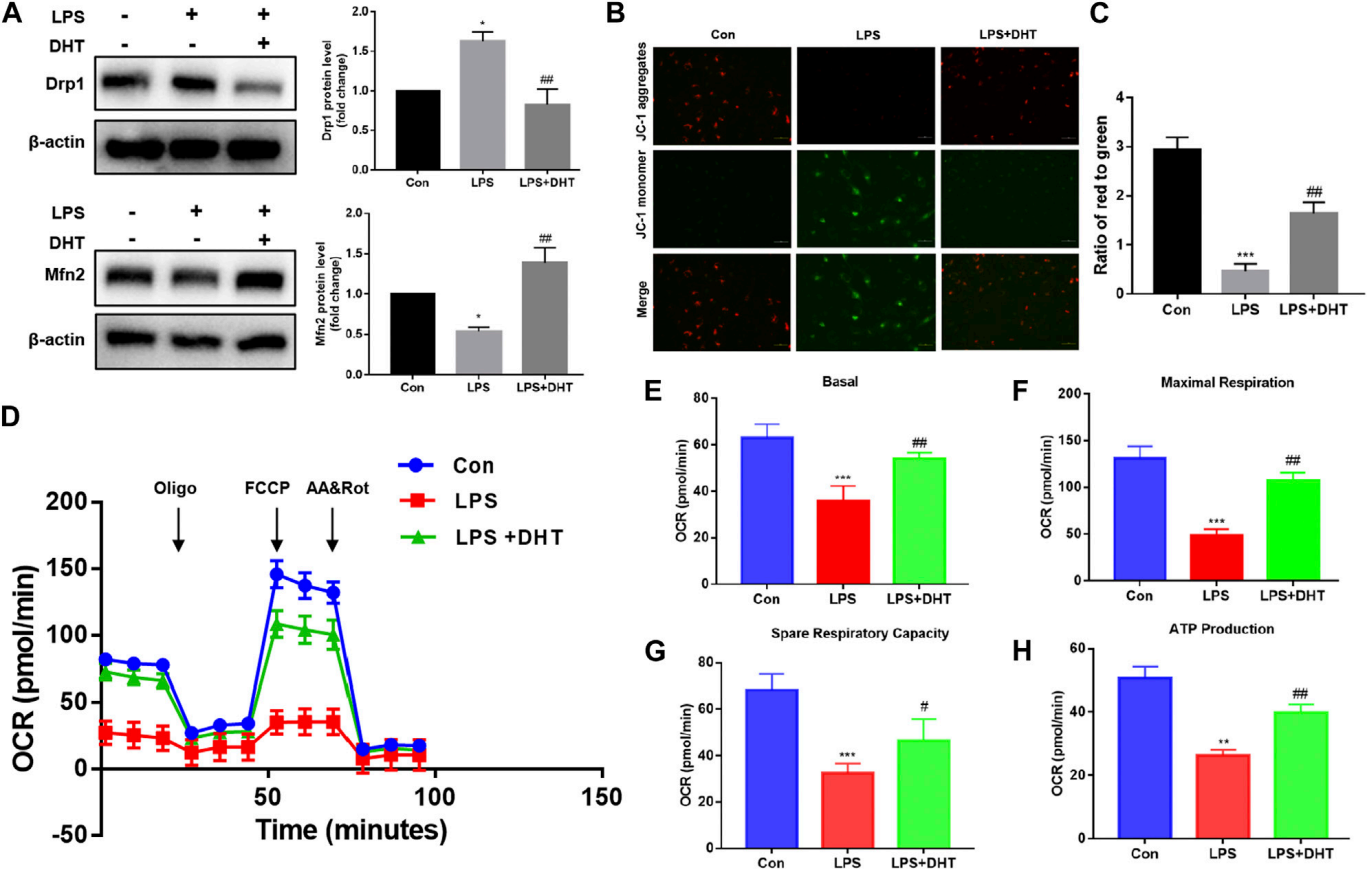
**(A)** Protein expression and quantitative analysis of Mfn2 and Drp1 were assessed by Western blot analysis and normalized to the intensity of *β*-actin (n = 3). ^*^ *p* < 0.05, ^**^ *p* < 0.01 vs. Con, ^#^ *p* < 0.05, ^##^ *p* < 0.01 vs. LPS. **(B)** MMP was detected using a JC-1 fluorescent probe. Red fluorescence represents aggregates, while green fluorescence represents monomer. Scale bars: 50 μm. **(C)** Quantitative data of the red to green fluorescence intensity ratio in HK-2 cells (n = 3). ^***^ *p* < 0.001 vs. Con, ^##^ *p* < 0.01 vs. LPS. **(D)** OCR of HK-2 cells after oligomycin (1.5 µM), FCCP (1.0 µM), and rotenone/antimycin A (R&A at 0.5 µM) treatments was recorded. A mitochondrial stress test was conducted to detect mitochondrial energy metabolism and respiratory function in LPS-induced HK-2 cells treated with or without DHT. The background is the untreated cell. **(E–H)** Basal respiration, maximal respiration, spare respiration capacity, and ATP production in each group (n = 3). ^**^ *p* < 0.01, ^***^ *p* < 0.001 vs. Con, ^#^ *p* < 0.05, ^##^ *p* < 0.01 vs. LPS.

Previous studies showed that COX2 protein expression was closely related to mitochondria quality control. The correct process of mitochondrial biosynthesis provides sufficient ATP for cells and plays an important role in cell survival. As a major site of energy metabolism, mitochondria play a vital part in regulating oxidative injury. Mitochondrial fusion and fission are always in dynamic equilibrium, which is regulated by Mfn2 and Drp1. To further confirm the effect of DHT on mitochondrial dynamics, the expression of Mfn2 and Drp1 was evaluated by Western blot. Our data demonstrated that LPS decreased mitochondrial fusion and increased fission, and pretreatment with DHT reversed this phenomenon (Figure 7A). A reduction in the mitochondrial membrane potential (MMP) showed impaired mitochondrial function. A reduction of MMP was observed in LPS-treated HK-2 cells compared with the control as shown by an increase in JC-1 monomer and a decrease in JC-1 aggregation. However, MMP was restored when LPS-induced HK-2 cells were pretreated with DHT (Figures 7B, C). Mitochondrial oxidative phosphorylation was measured using an XFe24 Seahorse Analyzer and a mitochondrial stress test kit. The results showed that LPS inhibited the oxidative phosphorylation and mitochondrial respiration of HK-2 cells, including the oxygen consumption rate (OCR) (Figure 7D), basic respiration (Figure 7E), maximum respiration (Figure 7F), spare respiration capacity (Figure 7G), and ATP production (Figure 7H), but DHT pretreatment could improve the mitochondrial respiratory capacity in HK-2 cells subjected to LPS. These data illustrate the protective effect of DHT against the imbalance of LPS-induced mitochondrial dynamics and respiratory dysfunction.

### DHT reduced apoptosis in LPS-induced HK-2 cells

**FIGURE 8:**
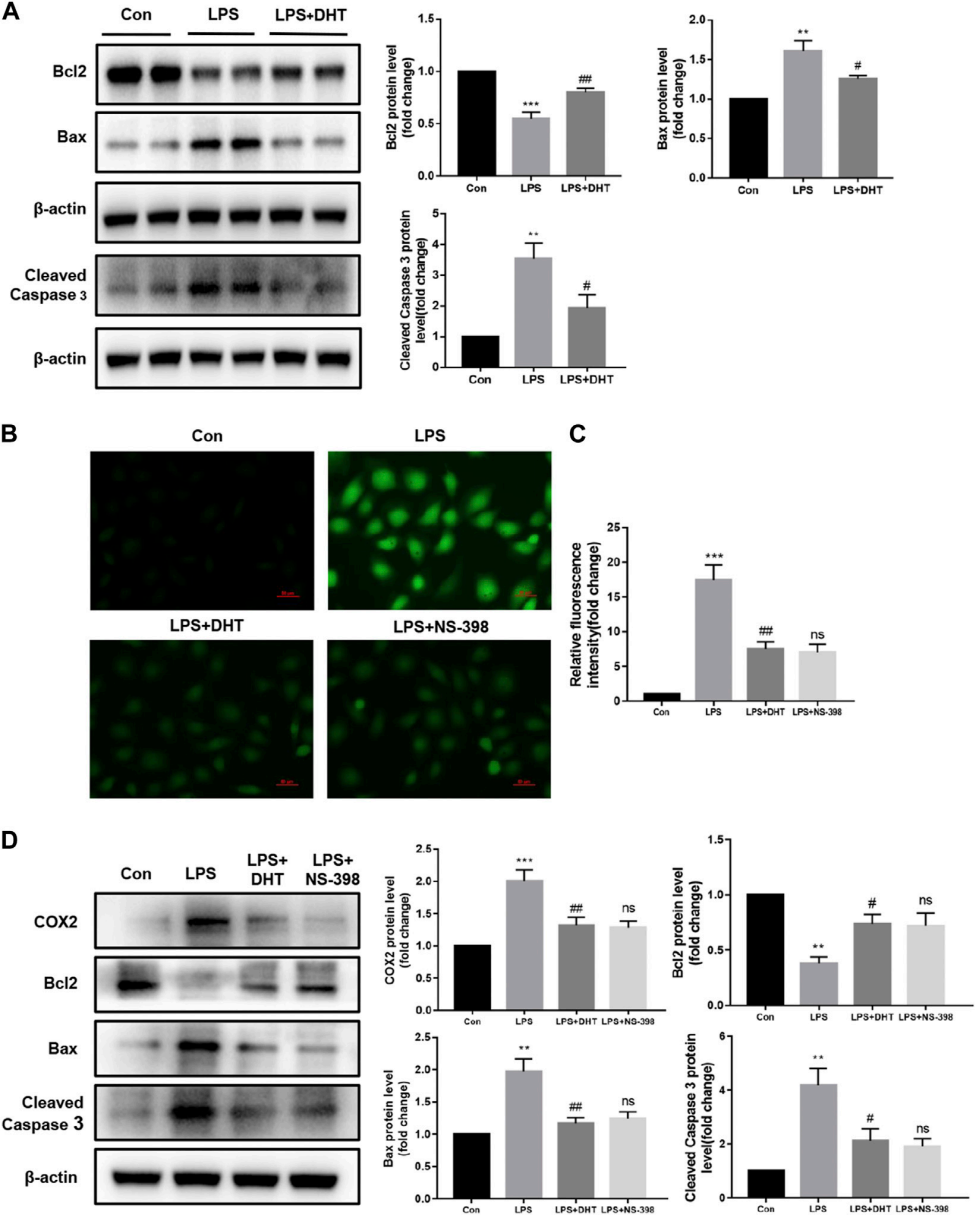
DHT alleviated LPS-induced apoptosis in HK-2 cells. **(A)** Representative Western blot and quantitative data showing the effect of DHT on the expression levels of Bcl2, Bax, and cleaved caspase 3 in LPS-induced HK-2 cells (n = 3). ^**^ *p* < 0.01, ^***^ *p* < 0.001 vs. Con, ^#^ *p* < 0.05, ^##^ *p* < 0.01 vs. LPS. **(B)** Representative image of ROS measured by fluorescence microscopy in each group. Scale bars: 50 μm. **(C)** Quantitative data of ROS fluorescence intensity in HK-2 cells (n = 3). ^***^ *p* < 0.001 vs. Con, ^##^ *p* < 0.01 vs. LPS, ns vs. LPS + DHT. **(D)** Representative Western blot and quantitative data showing the level of COX2 and apoptosis-related proteins in Con, LPS, LPS + DHT, and LPS + NS-398 (n = 3). ^**^ *p* < 0.01, ^***^ *p* < 0.001 vs. Con, ^#^ *p* < 0.05, ^##^ *p* < 0.01 vs. LPS, ns vs. LPS + DHT.

Mitochondrial dysfunction acts as an initiator in apoptosis during SI-AKI. Combined with the previous KEGG enrichment analysis, the effect of DHT on the LPS-induced HK-2 cell apoptosis pathway was further verified. Western blot results showed that after a 24-h treatment with 5 ug/ml LPS, the protein expression of apoptosis-related indicator Bcl2 decreased while Bax and cleaved caspase 3 increased. However, the LPS-induced changes in Bcl2, Bax, and cleaved caspase 3 were attenuated after pretreatment with DHT for 1 h (Figure 8A). To verify the effect of inhibiting COX2 on LPS-induced oxidative stress and apoptosis in HK-2 cells, the COX2 selective inhibitor NS-398 (5 μM) was used to treat cells. The effect of DHT and NS-398 on ROS production in LPS-treated HK-2 cells was determined by DCFH-DA. The results showed that DHT and NS-398 suppressed LPS-induced ROS accumulation (Figures 8B, C). Western blot results showed that DHT and NS-398 reversed the LPS-induced increase in COX2 and the expression of apoptosis-related proteins Bax, Bcl2, and cleaved caspase 3 in HK-2 cells (Figure 8D). These results demonstrate that DHT restrained LPS-induced apoptosis.

## Discussion

The immune and inflammatory responses caused by sepsis leads to multi-organ failure and death (Rudd et al., 2020). As an early event of sepsis, kidney injury is a common complication of hospitalized and critically ill patients (Poston and Koyner, 2019). SI-AKI is clinically characterized by severe infection with rapidly declining renal function. Renal failure leads to the accumulation of toxic substances in the body and is a major cause of death in critically ill patients. The resulting mortality rate is up to 70% and poses a great challenge for clinical treatment (Uchino et al., 2005). However, other than hemodialysis, there is an insufficiency of effective drugs to counteract renal tissue damage and renal function decline caused by sepsis. The complex pathophysiological process of sepsis, microcirculation disturbance, the inflammatory response, and renal tubular epithelial cell injury are considered the main causes of AKI (Peerapornratana et al., 2019). Sepsis promotes the release of inflammatory cytokines, enrolls immune cells to clear bacteria, and activates defense mechanisms against infection (Manrique-Caballero et al., 2021). Toll-like receptors on the surface of renal tubular epithelial cells recognize PAMPs and DAMPs, bind to TLRs to activate the NF-κB signaling pathway (Fry, 2012), upregulate COX2, and promote the expression of inflammatory cytokines IL-1β, IL-6, TNF-α, and MCP-1 (Novak and Koh, 2013). In addition, microvascular dysfunction further reduces renal blood perfusion and the blocking of the mitochondrial electron transport chain leads to the inhibition of cell oxidative phosphorylation. Reduced ATP production fails to provide energy to cells and intracellular ROS accumulation is a key component of oxidative stress (Nolfi-Donegan et al., 2020). Although there have been many studies on AKI, the underlying mechanism of SI-AKI has not been clarified, and it is an urgent need to find new targets and drugs for clinical treatment.

Recent studies have shown that COX2 is considered an important downstream effector in multiple organ injury in sepsis and plays a crucial role in inducing renal inflammatory reaction and oxidative stress (Simmons et al., 2004). The increased expression of COX2 in kidney tissue was previously observed in a mouse model of sepsis induced by CLP, and the inhibition of COX2 can alleviate sepsis-associated AKI (Ren et al., 2020; Guo et al., 2022). Studies have shown that COX2 is involved in the regulation of mitochondrial dynamics, which has been demonstrated in aging- and Parkinson’s-related neurodegenerative diseases. COX2 inhibitors maintain mitochondrial dynamical homeostasis by regulating the PGC-1α/NRF1/TFAM pathway. The inhibition of COX2 ameliorates nigrostriatal mitochondrial dysfunction to protect neurons after aging in rats, including the downregulation of Drp1 to reduce mitochondrial fission and the upregulation of Mfn2 expression to enhance mitochondrial fusion (Yan et al., 2021). Mitochondrial dysfunction is involved in the apoptosis of sepsis-induced renal tubular epithelial cells (Sun et al., 2019; Nezic et al., 2020). Abnormal mitochondrial division and reduced fusion make it difficult to form tight networks and cells are less resistant to oxidative stress and more susceptible to injury (Patil et al., 2014; Li et al., 2021). Previous reports suggest that reduced mitochondrial fusion and increased fission were detected in LPS-treated TECs, and cellular ROS aggregation triggered apoptosis (Jian et al., 2022), while the inhibition of mitochondrial damage was able to alleviate apoptosis (Zhang et al., 2018). These studies provide evidence that COX2 may be a potential therapeutic target for SI-AKI.

SM is a traditional and classical Chinese medicinal herb with a long history of use, rich natural resources, and wide distribution, and also has a broad prospect of development and utilization. It has several functions, including blood activation and anti-inflammatory and free radical scavenging, and is widely used in clinical practice. There are two main categories of active ingredients in SM: lipid-soluble danshenone diterpenoids and water-soluble phenolic acids, and it is crucial to select monomers with good stability and reproducibility for drug development (Su et al., 2015). The active ingredients of SM can protect the LPS-induced inflammatory reaction and oxidative stress. Salvianolic acid A can inhibit COX2 and significantly reduce the level of LPS-induced inflammatory mediators (Zeng et al., 2020). Our research suggests that DHT is the main active ingredient of SM, which belongs to tanshinone diterpenoids. Previous research found that DHT alleviated CCL4-induced acute liver injury by inhibiting the p38 and NF-κB signaling pathways (Yue et al., 2014). However, the effect and molecular mechanisms of DHT in the treatment of SI-AKI have not been reported.

Our research screened the main monomer components SM, a traditional Chinese medicine, and the potential targets for treating AKI through network pharmacological analysis. Molecular docking was used to assess the affinity relationship between DHT and COX2, and molecular dynamics simulations were used to validate their interaction patterns. We predict that DHT can combine with the active pocket of COX2 to induce protein degradation. Combined with experimental validation, we found that DHT had a protective effect against SI-AKI, and DHT downregulated the expression of COX2 in HK-2 cells induced by LPS in a dose-dependent manner. *In vivo*, the DHT pretreatment of mice alleviated CLP-induced renal histological injury, the loss of renal function, and inflammatory mediator release. *In vitro*, DHT pretreatment ameliorated LPS-induced HK-2 cell death, oxidative stress, and mitochondrial dysfunction, including stabilization of mitochondrial membrane potential, restoration of mitochondrial respiratory function, maintenance of mitochondrial dynamics, and the reversal of apoptosis-associated protein expression, to reduce the negative impacts of renal tubular injury.

However, the present study still has certain limitations. Although we performed a preliminary validation of DHT protection against SI-AKI *in vivo* and *in vitro*, and molecular docking and molecular dynamics simulations suggest that COX2 may be a potential target of DHT, we have not knocked down COX2 or used inhibitors to confirm that COX2 is the direct target of DHT in SI-AKI. However, this issue will be further explored in the future. In addition, more genes could be included in the study due to database updates, and other targets of action for DHT may exist.

## Conclusion

In summary, this study revealed the possible targets and mechanisms of DHT in alleviating SI-AKI through network pharmacological analysis, molecular docking, molecular dynamics simulation, and experimental verification. Our findings suggest that DHT prophylactically ameliorates the CLP surgery-induced decline in renal function, kidney tissue damage, and inflammatory factor release. *In vitro*, DHT downregulated LPS-induced COX2 expression in HK-2 cells, inhibited cell death and ROS production, and alleviated mitochondrial dysfunction and apoptosis. For the first time, we demonstrate the protective effect of DHT on sepsis-induced renal tubular epithelial cell injury and provide a new strategy for the clinical treatment of SI-AKI.

## Data Availability

The original contributions presented in the study are included in the article/Supplementary Material, further inquiries can be directed to the corresponding authors.

## Abbreviations

BATMAN, Bioinformatics Analysis Tool for Molecular Mechanism; BUN, urea nitrogen; CTD, Comparative Toxicogenomics Database; Con, control; CLP, cecal ligation and puncture; COX2, cyclooxygenase-2; DL, drug-likeness; DMEM/F12, Dulbecco’s Modified Eagle Medium/Nutrient Mixture; DHT, dehydromiltirone; F-12 FBS, fetal bovine serum; GO, Gene Ontology; HE, hematoxylin-eosin; HL, half-life; IL-1β, interleukin-1β; IL-6, interleukin-6; KEGG, Kyoto Encyclopedia of Genes and Genomes; LPS, lipopolysaccharide; MCC, maximal clique centrality; MCP-1, monocyte chemoattractant protein-1; MMP, mitochondrial membrane potential; OB, oral bioavailability; OCR, oxygen consumption rate; PDB, Protein Data Bank; PPI, protein-to-protein interaction; Scr, serum creatinine; SI-AKI, sepsis-induced acute kidney injury; ROS, reactive oxygen species; TCMID, Traditional Chinese Medicine Integrated Database; TCMSP, Traditional Chinese Medicine Systems Pharmacology; TNF-α, tumor necrosis factor-α.
