# Supplementary material for: Investigating the effect of dehydromiltirone on septic AKI using a network pharmacology method, molecular docking, and experimental validation
Source: Front Pharmacol. 2023 Mar 15;14:1145675. doi: 10.3389/fphar.2023.1145675 (PMC10050741; doi:10.3389/fphar.2023.1145675)
Supplement: Supplementary file 1 [file Table1.DOCX]

Supplementary Material

# Supplementary Table

# Supplementary Table 1. Primers Used in qRT-PCR.

| Name | Sequence (5′-3′) |
| --- | --- |
| β-actin | GTCATTCCAAATATGAGATGCGT |
|  | TGTGGACTTGGGAGAGGACT |
| IL-6 | CTGCAAGAGACTTCCATCCAG |
|  | AGTGGTATAGACAGGTCTGTTGG |
| IL-1β | GAAATGCCACCTTTTGACAGTG |
|  | TGGATGCTCTCATCAGGACAG |
| TNF-α | GGCTACTTGGGCTATTGTAAAGG |
|  | CAGTTTCTCCGACAACTTTCTCT |
| MCP-1 | TAAAAACCTGGATCGGAACCAAA |
|  | GCATTAGCTTCAGATTTACGGGT |

# Supplementary Table 2. Top 3 molecular docking affinity score

| **Molecule** | **Gene** | **Affinity** |
| --- | --- | --- |
| Dehydromiltirone | COX2 | -9.4 |
| Salviol | TGF-β1 | -7.4 |
| Miltirone I | IL6 | -6.4 |
